# Transcriptomic changes associated with heat-induced susceptibility in wheat plants to Hessian fly (Diptera: Cecidomyiidae)

**DOI:** 10.3389/fpls.2026.1794823

**Published:** 2026-05-05

**Authors:** Lieceng Zhu, Mohamed Maldani, Subhashree Subramanyam, Ming-Shun Chen, Rui Shi

**Affiliations:** 1 Plant Interactions Laboratory, Department of Biological and Forensic Sciences, Fayetteville State University, Fayetteville, NC, United States; 2 Crop Production and Pest Control Research Unit, USDA-ARS and Department of Entomology, Purdue University, West Lafayette, IN, United States; 3 Hard Winter Wheat Genetics Research Unit, USDA-ARS and Department of Entomology, Kansas State University, Manhattan, KS, United States; 4 Crop and Soil Sciences Department, North Carolina State University, Raleigh, NC, United States

**Keywords:** heat stress, hessian fly, susceptibility, transcriptomic changes, wheat

## Abstract

Wheat (*Triticum aestivum* L.) resistance to Hessian fly (HF, *Mayetiola destructor*) conferred by HF resistance (*R*) genes, is often vulnerable to heat stress. This study aims to investigate the molecular basis of heat-induced susceptibility in wheat to HF. We compared the resistant cultivar ‘Molly’, which carries *H13*, with its susceptible near-isogenic line ‘Newton’ to determine how elevated temperature alters phenotypic and molecular responses to HF infestation. Our phenotyping results showed that a single 24 h heat treatment at 30 °C was sufficient to compromise Molly’s resistance, resulting in more than 70% of plants becoming susceptible. Transcriptomic profiling revealed that resistant Molly exhibited stronger and broader defenses than Newton under normal temperature, whereas heat-stressed Molly displayed extensive transcriptional reprogramming resembling the naturally susceptible Newton wheat. Comparative analysis of transcriptomic profiles identified 74 genes that are consistently regulated across all susceptible states at 24 h after initial HF infestation, including heat-stressed Molly and Newton under both high and normal temperatures, but not in the resistant Molly under normal temperature. Functional annotation of these susceptibility-related genes in combination with previous findings suggest that susceptibility is likely associated with increased auxin-related activity, reduced salicylic acid (SA) and OPDA-associated defense signaling, and altered coordination of defense pathways that favor feeding-site establishment and redirecting host resource to developing HF larvae. The identification of susceptibility-associated candidate genes provides molecular targets for breeding wheat cultivars with more durable resistance under rising temperatures.

## Introduction

Wheat (*Triticum aestivum* L.) is a staple food crop of global importance. The Hessian fly (HF, *Mayetiola destructor*, Say) is one of the most destructive insect pests of wheat worldwide. HF attacks wheat plants at larval stage by feeding on the stem between leaf sheaths at the base of a seedling plant, causing stunted plants which die eventually, leading to substantial yield losses (Byers and Gallun, 1972; Ratcliffe and Hatchett, 1997). The most effective and environmentally-sound strategy for managing HF damage is the use of resistant wheat cultivars harboring specific resistance (*R*) genes (Ratcliffe and Hatchett, 1997; Chen et al., 2009). To date, over 37 HF resistance genes have been identified, with some being deployed in commercial wheat varieties (Xu et al., 2024). However, the *R*-gene mediated resistance is fragile under changing environmental conditions. Substantial evidence indicates that abiotic stresses, particularly heat stress, can severely compromise wheat’s ability to mount effective defenses against HF attack (Chen et al., 2014; Currie et al., 2014a; Cheng et al., 2018; Zhu et al., 2020; Subramanyam et al., 2025). Therefore, understanding the mechanism of wheat resistance against HF and the heat-stress induced loss of wheat resistance is of great importance to develop wheat cultivars with more durable insect resistance.

The mechanism of the *R*-gene mediated wheat resistance to HF has been well studied during the past two decades. Overall, resistance responses of wheat to HF infestation in incompatible interactions (resistant plant, dying HF larva) rely on rapid mobilization of plants’ chemical and energy resources including increase in degradation of lipid, sugar, and amino acids to provide substances and energy needed for synthesizing defense compounds, such as phenylpropanoids, flavonoids, and wax (Kosma et al., 2010; Zhu et al., 2012; Khajuria et al., 2013). In compatible interactions (susceptible plant, live HF larva), however, the expression levels of genes related to resource mobilization and synthesis of defense chemicals were reduced, especially at early time points during the wheat-HF interaction (Khajuria et al., 2013), as the larvae are able to manipulate plants resulting in the formation of nutritive tissue that provide the developing larvae a rich source of nutrients (Harris et al., 2006). Accumulation of phytohormones is also distinctively different between resistant and susceptible plants. High levels of 12-oxophytodienoic acid (OPDA) and salicylic acid (SA) accumulation occurred in HF feeding tissues of resistant wheat plants during the incompatible interaction, but high levels of auxin (IAA) accumulation occur in susceptible plants during the compatible interaction (Zhu et al., 2010). While extensive studies have elucidated the molecular mechanisms underlying wheat resistance and susceptibility to HF infestation (Williams et al., 2002; Sardesai et al., 2005; Giovanini et al., 2007; Liu et al., 2007; Zhu et al., 2008), the molecular mechanisms of heat-induced loss of wheat resistance to HF remain largely to be determined.

Previously, we studied the impact of heat stress on the phytohormone concentration, polar lipid concentration/composition, and changes in transcript abundance at HF feeding tissues in the wheat plants under normal and heat-stressed conditions using phytohormone/lipid profiling and RNA-sequencing (RNA-seq) to investigate the molecular basis underlying heat-induced loss of wheat resistance to HF infestation (Currie et al., 2014a, b; Cheng et al., 2018; Zhu et al., 2020; Yuan et al., 2022). Our findings indicate that heat stress can cause dramatic changes in primary and secondary metabolism, composition of membrane lipids and cell wall, plant hormones and other defense-related pathways. Exogenous application of phytohormones such as SA and OPDA significantly enhances HF resistance in wheat plants under heat conditions (Underwood et al., 2014; Cheng et al., 2018; Maldani et al., 2024). Our findings in combination with previous reports suggested that heat stress impairs pathways in wheat plants that produce and mobilize resources needed for synthesizing defensive compounds and cell wall fortification against HF invasion. In the current study, we aimed to further elucidate systematically the complex molecular responses in wheat plants to HF infestation under heat stress, focusing on the common changes in gene expression in naturally susceptible and heat-induced susceptible plants. To this end, we used the resistant wheat cultivar ‘Molly’, which harbors the *H13* resistance gene and its susceptible near-isogenic line (NIL)’Newton’, for RNA-seq analyses. By analyzing the commonly regulated genes in naturally and heat-induced susceptible plants, we provide new insights into the mechanism of susceptibility and identified candidate genes potentially involved in heat-induced wheat susceptibility.

## Materials and methods

### Plant and insect material

The wheat cultivars Molly and Newton and a HF biotype GP (Great Plains) were used in this study. Molly and Newton are near isogenic lines (NILs) (Patterson et al., 1994). Molly possesses the HF resistance gene *H13* and is resistant to the avirulent HF biotype GP under room temperature or below (Underwood et al., 2014). Newton is susceptible to biotype GP.

### Plant preparation and infestation

Twenty Molly seeds were planted in each pot of 10-cm in diameter filled with potting mix (Scotts Miracle-Gro Company, Marysville, OH). The pots were placed in a growth chamber set at 20°C and 14:10 (day: night) photoperiod until most seedlings grew into 1.5 leaf stage. To infest, eight newly emerged female flies and two male flies were released onto plants confined within a cage covered with a piece of cheese cloth. Those female HFs would lay eggs on leaves of wheat seedlings. Those eggs would develop into larvae which crawl down to the base of the plants, hiding behind the first leaf sheath and attack the second leaf sheath. To determine the time when larvae began to attack plants, randomly selected plants were dissected and observed hourly under a dissection microscope beginning at 72 h following the release of adult HF. The time when HF larvae were first seen at the base of a plant was taken as the time for initial HF larval attack.

### Treatment, experimental design, and statistical analysis for phenotyping

Molly wheat seedlings infested with HF biotype GP were subjected to 30 °C heat stress for one day (24 h), two days (48 h), and four days (96 h), respectively. Wheat plants were initially grown and infested in a growth chamber set at 20 °C with 14 h:10 h (Light: Dark) cycle. At the time of initial HF attack, plants designated for heat stress were moved to a second growth chamber set at 30 °C, for 24 h, 48 h, or 96 h, respectively. The plants were returned to the original growth chamber and placed back in their original position within the same block (row) after the heat treatments. Control plants were maintained in the original growth chamber at 20 °C throughout the experiment.

The experiment was designed based on Randomized Complete Block Design (RCBD) with three biological replicates (one pot for each replicate of each treatment). To minimize potential environmental variation within the growth chamber, the chamber was divided into three rows, each corresponding to one biological replicate. Within each row, all treatments were included, and the position of the treatments were assigned randomly. Resistance and susceptibility of each plant was determined seven days after the initial attack (Currie et al., 2014b). For phenotyping, the number of dead and live larvae were counted for each plant of each replicate in each treatment. The percentage of susceptible plants and the percentage of live larvae were used to determine the level of resistance or susceptibility in each replicate of each treatment. The percentage of susceptible plants for each pot was calculated as ‘Number of susceptible plants/Total number of infested plants. A plant was rated as resistant if it contained all dead larva(e), but susceptible if it contained live larva(e). The percentage of live larvae per pot was calculated as ‘Number of live larvae/Number of total larvae’. The increased percentages of susceptible plants and live larvae indicate a higher level of susceptibility. The data were analyzed using the PROC GLM procedure of SAS software version 9.4 (SAS Institute Inc, 2013). Differences among treatments were evaluated using Tukey’s Honestly Significant Difference (HSD) test (α=0.05) to determine statistical significance.

### Treatment, experimental design, and sampling for RNA sequencing

The experiment for RNA-seq included 10 treatments (**Table 1**). For either Molly or Newton, treatments included uninfested controls, the infested plants under normal temperature or heat stress sampled at 24 h and 72 h after the initial HF attack, respectively. Heat stress treatment was applied by placing plants in a growth chamber set at 30 °C for 24 h or 72 h, respectively, starting at the time when HF larvae-initiated attacks on plants. The experiment was conducted in two Percival growth chambers (Perry, IA) following the Randomized Complete Block Design (RCBD) with three biological replicates. Within the same treatment, each pot is a replicate containing 10 seedlings. At the time of sampling, each wheat seedling was cut from its base. The second leaf sheath was carefully peeled off, and a10-mm section of each second leaf sheath was collected into a 1.5-ml Eppendorf tube filled with RNAlater (Thermo Fisher Scientific, Waltham, MA). For plants infested with HF, the second leaf sheaths were rinsed in water to remove HF larvae from infested plants and dried on a paper towel to remove excessive water before collecting the sample into a tube. Only plants containing 10 or more larvae were sampled. Samples were stored at -80 °C before shipping to Novogene (Sacramento, CA) for RNA extraction, library construction, sequencing, and basic bioinformatic analysis.

**Table 1:** Experimental treatments.

No.	Name	Description
1	MCK	Molly under normal temperature (20 °C), uninfested, sampled at 24 h after initial HF attack
2	M20C24H	Molly under normal temperature, infested, sampled at 24 h after initial HF attack
3	M30C24H	Molly under 24 h heat stress (30 °C), infested, sampled at 24 h after initial HF attack
4	M20C72H	Molly under normal temperature, infested, sampled at 72 h after initial HF attack
5	M30C72H	Molly under 72 h heat stress (30 °C), infested, sampled at 72 h after initial HF attack
6	NCK	Newton under normal temperature, uninfested, sampled at 24 h after initial HF attack
7	N20C24H	Newton under normal temperature, infested, sampled at 24 h after initial HF attack
8	N30C24H	Newton under 24 h heat stress (30 °C), infested, sampled at 24 h after initial HF attack
9	N20C72H	Newton under normal temperature, infested, sampled at 72 h after initial HF attack
10	N30C72H	Newton under 72 h heat stress (30 °C), infested, sampled at 72 h after initial HF attack

### RNA extraction, library construction, and sequencing

RNA was extracted using TRIzol Reagent (Thermo Fisher Scientific) following the manufacture’s instruction. Libraries were generated using NEBNext^®^ Ultra™ RNA Library Prep Kit for Illumina^®^ (New England Biolabs, Ipswich, MA) following manufacturer’s recommendations. Briefly, mRNA was purified from total RNA using poly-T oligo-attached magnetic beads. Fragmentation was carried out using divalent cations under elevated temperature in NEBNext First Strand Synthesis Reaction Buffer 5X. First strand cDNA was synthesized using random hexamer primer and M-MuLV Reverse Transcriptase (RNase H). Second strand cDNA synthesis was performed using DNA Polymerase I and RNase H followed by a round of purification, terminal repair, A-tailing, ligation of sequencing adapters, size selection and PCR enrichment. Library concentration was first quantified using a Qubit 2.0 fluorometer (Life Technologies, Carlsbad, CA) and then diluted to one ng/μl before checking insert size on an Agilent 2100 system (Agilent Technologies, Santa Clara, CA). Libraries were sequenced using an Illumina HiSeq-PE150 sequencing platform.

### Quality control, sequence alignment, and quantification of transcript abundance

Raw reads in FASTQ format were filtered to remove reads containing adapters or reads of low quality to produce clean reads. Paired-end clean reads were then mapped to the *T. aestivum* reference genome version ‘ensembl_57_triticum_aestivum_iwgsc_toplevel’ (https://www.novogene.com/us-en/Reference/ReferenceForSpecies/index.html) using HISTAT (v2) (Mortazavi et al., 2008). Only uniquely mapped reads were used for further read counts per gene, normalization of read counts, and gene expression analyses. HTSeq (v 0.6.1) was used to generate read counts for each gene (Anders et al., 2015).

### Differential expression and functional analyses

Differential expression analyses were performed on normalized read counts of pairwise treatments using DESeq2 R package (Love et al., 2014). The resulting *p*-values were adjusted using Benjamini and Hochberg’s approach for controlling the false discovery rate. Genes with an adjusted *p*-value (q value) < 0.05 and an absolute log2 fold change ≥ 1were considered differentially expressed. Cluster analysis of gene expression was based on the log_2_ (FPKM + 1) value of all 30 samples of 10 experimental treatments. GO and KEGG enrichment analyses were conducted using Cluster Profiler (Yu et al., 2012).

### qRT-PCR validation of transcript abundance

To validate the RNA-seq data, 10 up-regulated and 10 down-regulated genes between two treatments M30C72H (heat stressed) and M20C72H (non-heat stressed) (**Table 1**) were randomly selected for quantitative real-time reverse transcription polymerase chain reaction (qRT-PCR) analysis. Gene-specific primers (**Supplementary Table 1**) were designed using Primer Express 3.0 (Applied Biosystems, Waltham, MA). Gene expression analysis was performed using QuantStudio 6 Pro (Applied Biosystems) with PowerUp SYBR master mix (Applied Biosystems) according to the manufacturer’s protocol. Each reaction was carried out in triplicate with the ubiquitin (UBQ) gene as the endogenous control for normalization. No-template control was included in each assay. The data were collected from three biological replicates, each containing three technical replicates. Amplification of a single product was confirmed through melt-curve analysis. Transcript abundance was quantified using the Relative Standard Curve Method (Applied Biosystems User Bulletin 2, ABI PRISM 7700 Sequence Detection System). Significant differences were determined by analysis of variance (ANOVA) with SAS software version 9.4 (SAS Institute Inc, 2013) according to Hargarten et al. (2017). The data is represented as log_2_FC and the differences between the heat stressed and the non-heat stressed samples were considered statistically significant if the *p*-value associated with the contrast was < 0.05.

## Results

### Impact of different heat doses on wheat resistance and HF larval survival

**Figure 1:**
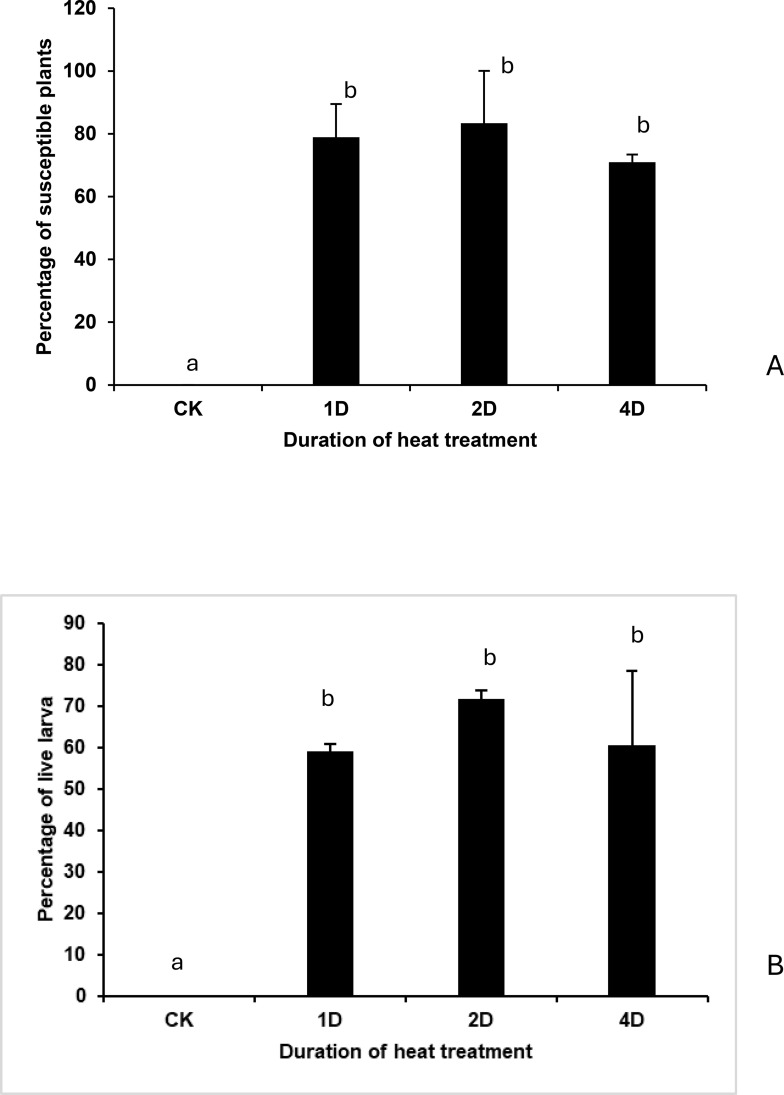
Impact of different doses of heat treatments on the percentage of susceptible plants **(A)** and live larvae **(B)** in the HF-infested molly plants. CK- control without heat stress at 20 °C; plants were heat-stressed for one day (1D), two days (2D) and four days (4D), after initial larval attack. Treatments marked with the same letter are not statistically significant.

Under normal temperature of 20 °C, Molly plants exhibited complete resistance with no live larvae in all tested plants (**Figure 1**). Heat treatments at 30 °C for one day, two days, and four days, however, reduced plants resistance significantly, resulting in an average of 71 to 83% susceptible plants (**Figure 1A**; F = 24.09, df=3, *p* = 0.0021) and 59 to 72% of live HF larvae (**Figure 1B**; F = 9.89, df = 3, *p* = 0.0152) at seven days after initial HF attacks on the plants. No significant difference was found in plant susceptibility among different heat doses.

### Overview of RNA-seq analysis

**Figure 2:**
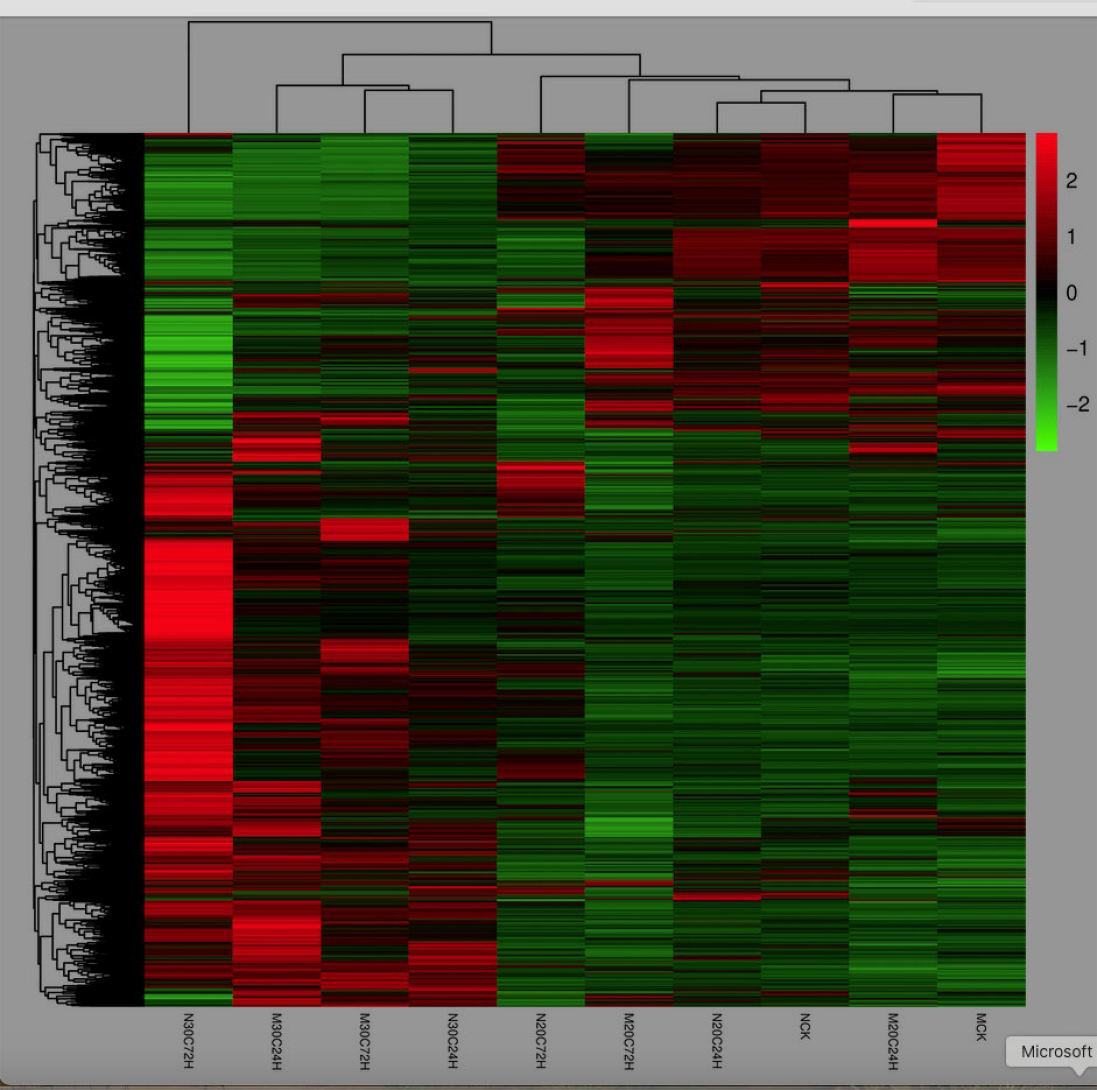
Heat map showing expression profile of genes in molly and newton plants under different treatments, including the uninfested molly (MCK), uninfested Newton (NCK), infested Molly (M20C24H), infested Newton (N20C24H) under normal temperature at 24 h after initial larval attack, Molly (M30C24H) and Newton (N30C24H) plants treated with the combination of infestation and heat stress at 30 °C for 24 h, infested Molly (M20C72H) and infested Newton (N20C72H) plants under normal temperature at 72 h after initial larval attack; and Molly (M30C72H) and Newton (N30C72H) treated with the combination of infestation and heat stress at 30 °C for 72 h.

An average of 69.4 million of 2 × 150 paired-end raw reads per sample, ranging from 61.1 to 88.5 million, were obtained (**Supplementary Table 2**). Read counts between the replicates of each treatment are highly correlated with R^2^ ≥ 95.7% for the same treatment (**Supplementary Table 3**), indicating high repeatability of the sequencing data. An average of 68.4 million (98.6%) clean reads were obtained after quality trimming (**Supplementary Table 2**). Overall, 91.3% of clean reads were mapped to the *T. aestivum* reference genome, and 86.4% were uniquely mapped (**Supplementary Table 2**). A total of 31,218 informative genes were identified. Based on the Log10 (FPKM + 1) value of all informative genes, a cluster heat map was generated, showing a distinctive transcript expression pattern among the 10 treatments used in this study (**Figure 2**).

### qRT-PCR validation of transcript abundance

**Figure 3:**
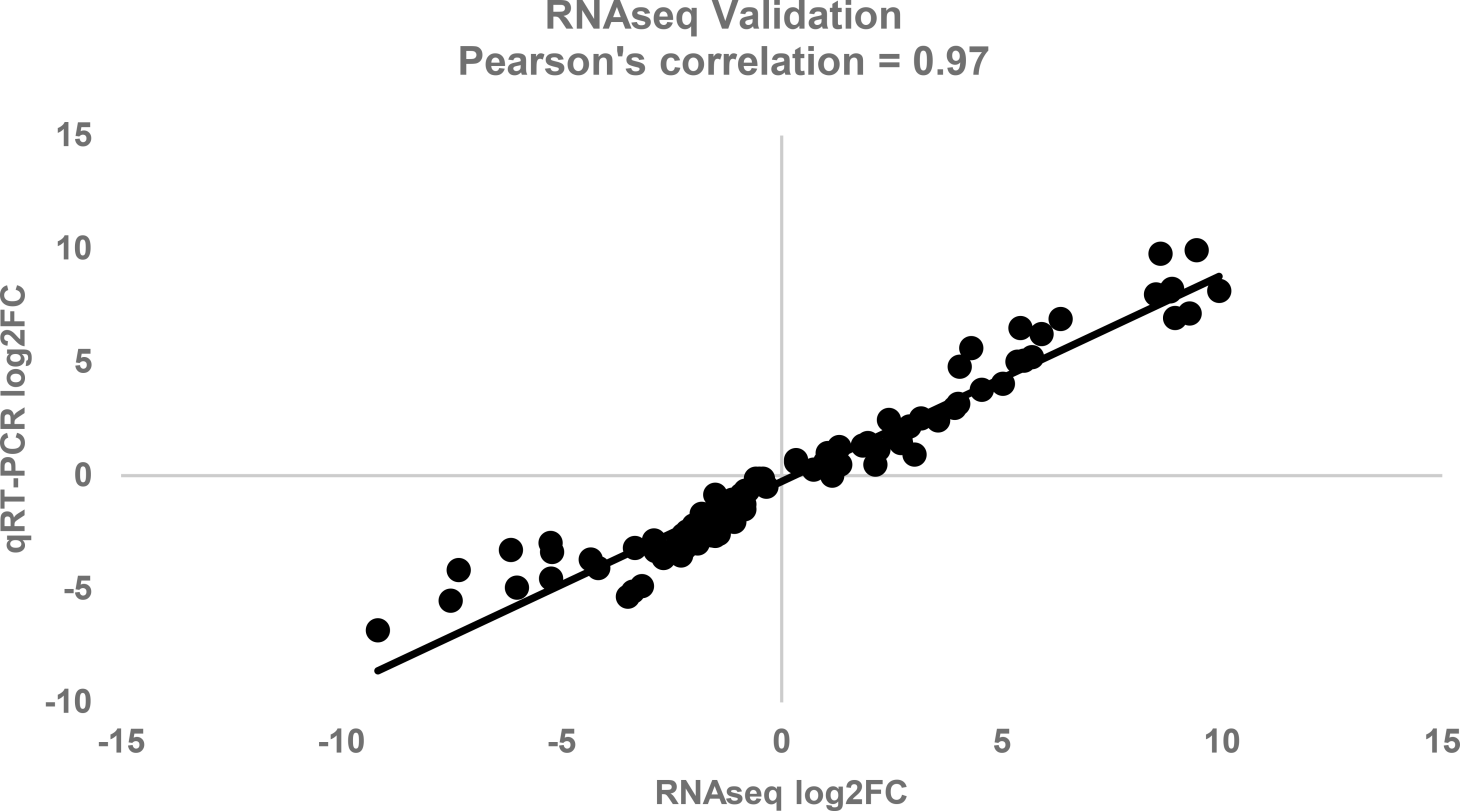
Correlation between log_2_ fold change between the qRT-PCR and RNA-Seq results for 10 upregulated and 10 downregulated differentially expressed genes (DEGs) observed in RNA-Seq experiments.

A total of 10 upregulated and 10 downregulated genes between the two treatments M30C72H and M20C72H (**Table 1**) were selected for qRT-PCR validation of the RNA-seq results. The Pearson correlation coefficient between the log_2_ fold changes obtained from RNA-seq and qRT-PCR was 0.97, indicating a strong positive correlation (**Figure 3**).

### Stronger responses to infestation in Molly at early wheat-HF interaction

**Figure 4:**
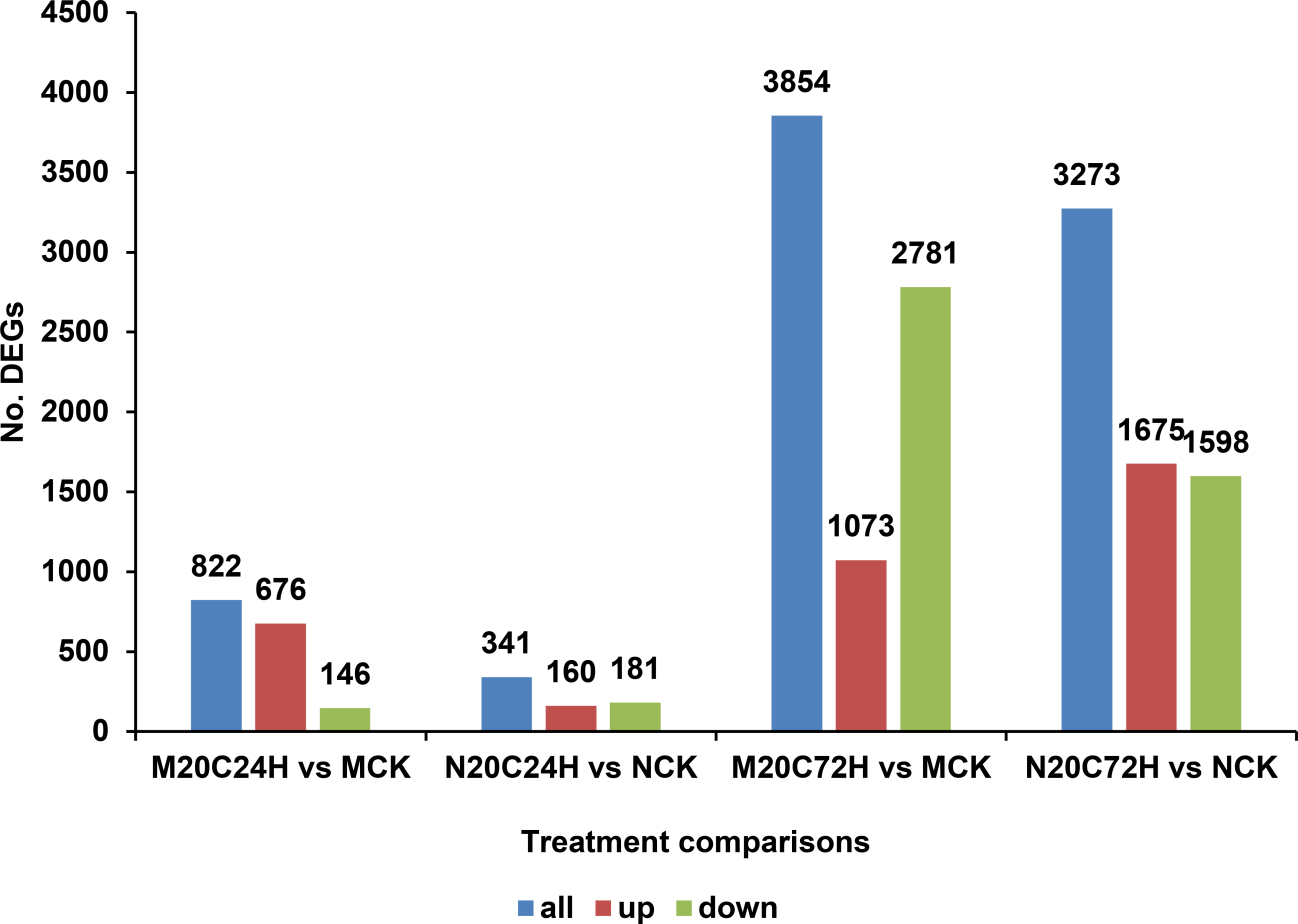
Number of total (all) upregulated (up), and downregulated (down) differentially expressed genes (DEGs) caused by HF infestation under normal temperature (20 °C) in molly and newton at 24 and 72 h after initial larval attack, respectively, compared to their respective uninfested control plants. M20C24H: Infested Molly at 24 h; M20C72H: Infested Molly at 72 h; MCK: Uninfested Molly controls at normal temperature; N20C24H: Infested Newton at 24 h; N20C72H: Infested Newton at 72 h; NCK: Uninfested Newton controls at normal temperature.

Our results indicated that HF infestation caused a stronger response in the resistant Molly than that in the susceptible Newton at the earlier point after fly attack. At 24 h after the initial attack of HF in plants under normal temperature, the number of differentially expressed genes (DEGs) in the resistant Molly is 2.4 times higher than that in the susceptible Newton. Most of these DEGs were upregulated by HF infestation. In contrast, the number of upregulated and downregulated DEGs were roughly the same in the susceptible Newton plants (**Figure 4**). The differences in the transcript expression in terms of the numbers of DEGs between Molly and Newton diminished at later time points. At 72 h, for example, the total numbers of DEGs were similar in both Molly and Newton plants (**Figure 4**).

KEGG analyses indicated that seven pathways were significantly enriched in Molly while four pathways were significantly enriched in Newton (**Tables 2**–**4**) at 24 h under normal temperature after initial HF larval attack. Of these enriched pathways, phenylalanine and tryptophan metabolism were enriched in both infested Molly and Newton plants compared to their respective controls. Phenylalanine metabolism plays a crucial role in plant defense against parasites, primarily through the phenylpropanoid pathway (Dixon et al., 2002; Yadav et al., 2020). The strong enrichment of phenylalanine metabolism with 17 upregulated genes in the resistant Molly but mild enrichment with five upregulated genes in the susceptible Newton plants suggest that the defensive response against HF in Molly is stronger than that in Newton (**Table 2**). Tryptophan metabolism, however, was enriched with all upregulated genes in Molly but all downregulated genes in Newton after HF attack (**Table 2**). Tryptophan metabolism contributes to plant defense against insects by producing a diverse array of defensive secondary metabolites and by playing a crucial role in regulating growth-defense trade-offs (Guo et al., 2022). The differential enrichment of tryptophan metabolism in Molly and Newton suggests that the upregulated tryptophan metabolism pathway may be critical to wheat resistance against HF infestation. Additionally, linoleic acid metabolism, α-linolenic acid metabolism, monoterpenoid biosynthesis, and ABC transporters are uniquely enriched in Molly with most or all upregulated genes, and carbon fixation in photosynthetic organisms is enriched with almost all downregulated genes in Molly (**Table 3**). The up-enrichment of these pathways was likely associated with stronger defense responses of wheat plants against HF infestation at early stage of plant-insect interactions in the resistant Molly plants. The down enrichment of carbon fixation in photosynthetic organisms is consistent with previous findings that HF infestation downregulates genes involved in carbohydrate metabolism during the incompatible interactions (Zhu et al., 2008). In the susceptible Newton, pathways for amino sugar and nucleotide sugar metabolism were enriched mostly by upregulated genes and flavonoid biosynthesis was enriched with all downregulated genes (**Table 4**). Flavonoids are secondary compounds that can act as feeding deterrents or interfere with insect growth and development (Ramaroson et al., 2022). The enrichment of flavonoids biosynthesis with downregulated genes in Newton suggests that production of antifeedants might be suppressed in the susceptible plants.

**Table 2:** Commonly enriched pathways in molly (M20C24H vs MCK) and newton (N20C24H vs NCK) at 24 h after initial HF attack under normal temperature.

KEGG ID	Description	Up		Down		Gene ratio		Background ratio	
		M	N	M	N	M	N	M	N
osa00360	Phenylalanine metabolism	17	5	0	0	17/203	5/72	139/14401	137/14488
osa00380	Tryptophan metabolism	9	0	0	5	9/203	5/72	199/14401	194/14488

Up, Gene counts of upregulated genes; Down, Gene counts of downregulated genes; M, Molly; N, Newton.

**Table 3:** Uniquely enriched pathways in molly (M20C24H vs MCK) at 24 h after initial HF attack under normal temperature.

KEGG ID	Description	Up	Down	Gene ratio	Background ratio
osa00591	Linoleic acid metabolism	13	0	13/203	55/14401
osa00592	α-Linolenic acid metabolism	11	0	11/203	144/14401
osa00902	Monoterpenoid biosynthesis	5	0	5/203	32/14401
osa02010	ABC transporters	10	1	11/203	205/14401
osa00710	Carbon fixation in photosynthetic organisms	1	10	11/203	214/14401

Up, Gene counts of upregulated genes; Down, Gene counts of downregulated genes.

**Table 4:** Uniquely enriched pathways in newton (N20C24H vs NCK) at 24 h after HF attack under normal temperature.

KEGG ID	Description	Up	Down	Gene ratio	Background ratio
osa00520	Amino sugar and nucleotide sugar metabolism	7	2	9/72	363/14488
osa00941	Flavonoid biosynthesis	0	4	4/72	115/14488

Up, Gene counts of upregulated genes; Down, Gene counts of downregulated genes.

### Greater impact of combined heat stress and HF infestation on Molly plants

**Figure 5:**
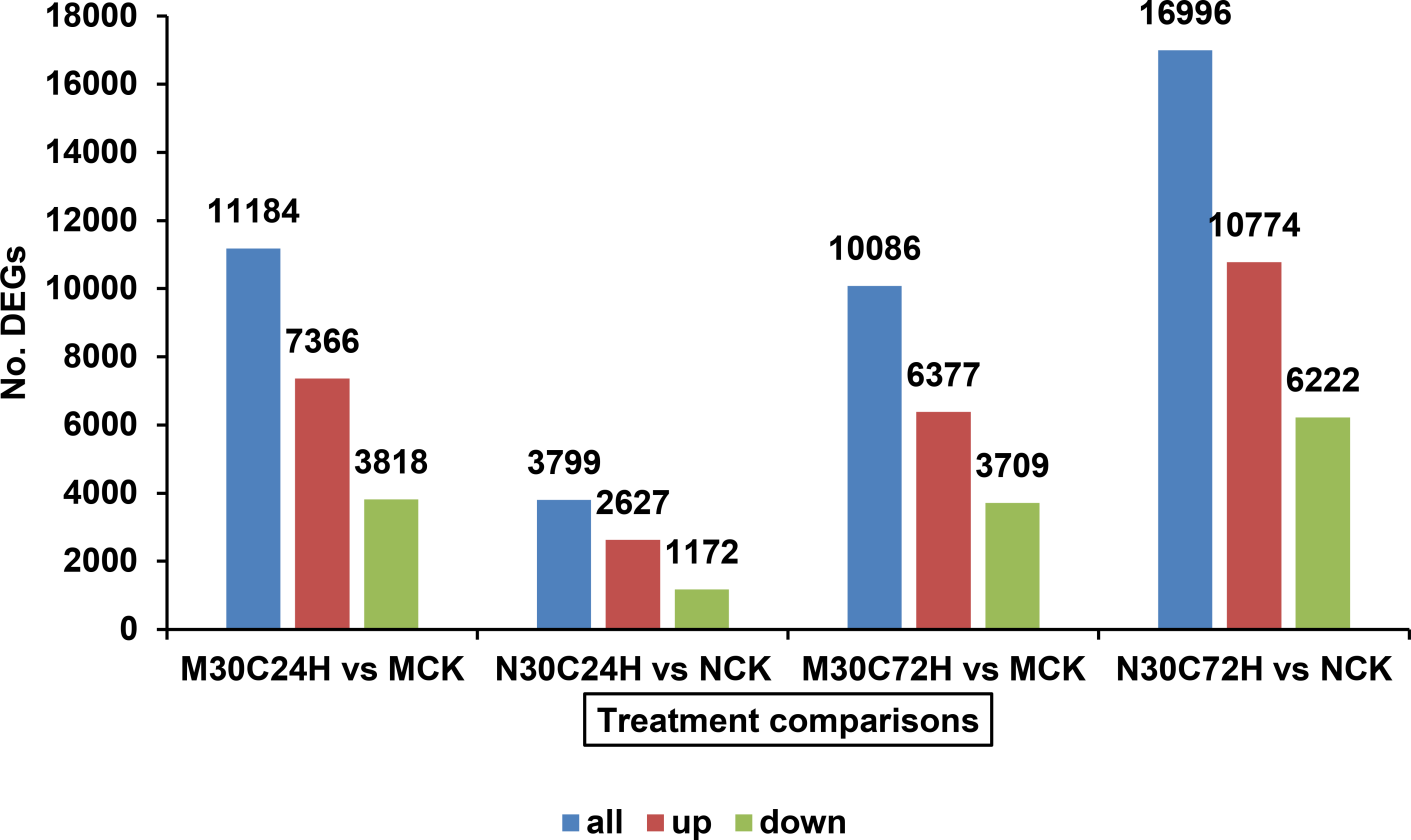
The numbers of total (all), upregulated (up) and downregulated (down) differentially expressed genes (DEGs) induced by the combination of HF infestation and 24 or 72 h heat stress (30 °C) in Molly and Newton plants. M30C24H: Infested Molly under 24 h heat stress; M30C72H: Infested Molly under 72 h heat stress; MCK: Uninfested Molly (control) at normal temperature; N30C24H: Infested Newton under 24 h heat stress; N30C72H: Infested Newton under 72 h heat stress; NCK: Uninfested Newton (control) at normal temperature.

At 24 h after the initial HF attack, the combination of heat stress and HF infestation triggered a much stronger transcriptional response in Molly compared to that in Newton (**Figure 5**). A total of 11,184 DEGs were identified in Molly plants as compared to the control, which is nearly three times more than that observed in Newton under similar conditions. In both cultivars, the majority of DEGs were upregulated at 24 h after the initial HF attack, however, at 72 h, the number of DEGs in Molly is considerably less than that in Newton (**Figure 5**). The stronger early response in Molly may reflect the substantial impact of heat stress on its metabolic processes, as heat compromises its otherwise effective resistance to HF (**Figure 1**). Regardless of timing or cultivar, the combined heat stress and HF infestation exert a much stronger effect on transcript abundance than that by HF infestation alone (**Figures 4**, **5**). A direct comparison between Molly plants exposed to both heat stress and HF infestation (M30C24H) and those only subjected to HF infestation under normal temperature at 24 h after the initial HF attack (M20C24H) resulted in 10,321 DEGs, with 6,878 genes upregulated and 3,443 downregulated, which highlight a substantial shift in metabolism due to heat stress. Gene Ontology (GO) term analysis identified 294 significantly enriched terms spanning biological processes, cellular components, and molecular functions (**Supplementary Table 4**). Collectively, these findings demonstrate that heat stress caused profound transcriptional reprogramming in the resistant Molly, leading to the compromised resistance to HF infestation.

### DEGs associated with natural susceptibility and heat-induced susceptibility

To identify DEGs associated with heat-induced susceptibility to HF larval infestation on Molly, we conducted a comparative transcriptomic analysis with samples derived from resistant and susceptible plants, respectively, at 24 h after the initial HF attack. Comparisons were made between 1) resistant Molly subjected to both heat stress and HF infestation (M30C24H vs. MCK); 2) susceptible Newton subjected to HF infestation at 20 °C (N20C24H vs. NCK); and 3) susceptible Newton subjected to both heat stress and HF infestation (N30C24H vs. NCK). This analysis yielded 97 shared DEGs between the heat-induced susceptible Molly (M30C24H vs. MCK) and the naturally susceptible Newton (N20C24H vs. NCK) under normal temperature. None of these 97 DEGS were observed in the resistant Molly under normal temperature (M20C24H vs. MCK). Further analysis indicates that 94 of those 97 DEGs were also caused by the combination of heat stress and HF infestation in the susceptible Newton at 24 h after the initial HF attack (N30C24H vs. NCK). Such results suggest that these genes are likely associated with susceptibility responses in Newton under both normal temperature and heat stress, as well as in Molly under heat stress. Of these 94 DEGs, 74 genes were successfully annotated, comprising 63 upregulated and 11 downregulated genes (**Tables 5**–**12**). These co-regulated genes represent a common susceptibility signature and can be functionally grouped into different categories.

**Table 5:** Co-upregulated phytohormone-related genes in susceptible and heat-stressed conditions at 24 h after initial HF attack.

Gene ID	Fold change			Gene description
	M30C24H vs MCK	N20C24H vs NCK	N30C24H vs NCK	
TraesCS4D02G069100	488.8	16.5	360.8	Auxin-repressed protein
TraesCS4B02G070300	450.3	19.8	389.0	Auxin-repressed protein
TraesCS4A02G245100	225.3	28.7	330.4	Auxin-repressed protein
TraesCS3A02G159200	2.5	5.6	4.5	Auxin responsive factor
TraesCS5B02G381800	2.8	2.3	2.3	Auxin-responsive protein
TraesCS5D02G388200	2.8	2.7	3.1	Auxin-responsive protein
TraesCS7A02G315300	5.5	3.8	5.7	Auxin-responsive protein
TraesCS6B02G411000	5.5	18.2	9.3	Auxin-responsive protein
TraesCS6D02G357500	6.7	7.3	4.2	Auxin-responsive protein
TraesCS6A02G373300	9.4	8.4	4.7	Auxin-responsive protein
TraesCS2D02G191800	9.8	8.9	14.0	Indole-3-acetic acid-amido synthetase GH3.8
TraesCS2B02G210600	16.4	11.4	14.4	Indole-3-acetic acid-amido synthetase GH3.8
TraesCS2A02G183900	8.7	20.5	23.2	Indole-3-acetic acid-amido synthetase GH3.8
TraesCS1A02G320200	7.7	6.2	4.3	Probable indole-3-acetic acid-amido synthetase GH3.4
TraesCS4D02G363600	9.2	8.7	9.3	VAN3-binding protein/Auxin canalization
TraesCS5A02G540300	3.0	6.7	4.4	VAN3-binding protein/Auxin canalization
TraesCS5A02G540500	7.1	13.5	9.7	VAN3-binding protein/Auxin canalization
TraesCS4B02G298600	8.6	73.1	52.3	Flavin-containing monooxygenase
TraesCS7B02G162100	6.9	66.1	144.4	Ethylene-responsive transcription factor ABR1/AP2 domain
TraesCS5D02G041100	3.5	6.0	23.6	Lipoxygenase, chloroplastic
TraesCS5B02G500700	4.4	5.5	4.9	DMR6-LIKE OXYGENASE 1
TraesCS2D02G392900	3.9	18.6	15.8	DMR6-LIKE OXYGENASE 1

M30C24H vs MCK: DEGs in the infested Molly plants under heat stress.

N20C24H vs NCK: DEGs in the infested Newton under normal temperature.

N30C24H vs NCK: DEGs in the infested Newton under heat stress.

**Table 6:** Co-upregulated genes encoding transcription factors and cell regulators in susceptible and heat-stressed conditions at 24 h after initial HF attack.

Gene ID	Fold change			Gene description
	M30C24H vs MCK	N20C24H vs NCK	N30C24H vs NCK	
TraesCS1D02G300900	2.4	6.2	10.5	WRK70_ Probable WRKY transcription factor 70
TraesCS2B02G517400	3.2	2.9	3.6	WRK70_SOLLC WRKY DNA-binding transcription factor 70
TraesCS1D02G300700	4.4	3.3	6.4	WRKY transcription factor 1
TraesCS2A02G489500	3.0	2.8	4.4	WRKY45-like transcription factor
TraesCS2D02G100200	7.3	13.6	10.7	WUSCHEL-related homeobox 7
TraesCS7B02G286300	7.8	5.3	8.0	B-box zinc finger protein
TraesCS1D02G398700	9.1	56.0	71.2	Cell number regulator 2
TraesCS2B02G437400	5.4	3.4	4.4	Probable LRR receptor-like serine/threonine-protein kinase
TraesCS5D02G398800	2.5	19.6	7.8	Probable LRR receptor-like serine/threonine-protein kinase
TraesCS2B02G222900	4.5	2.7	4.9	G-type lectin S-receptor-like serine/threonine-protein kinase
TraesCS3B02G596900	28.3	17.8	16.1	Wall-associated receptor kinase 3
TraesCS5B02G449200	3.0	6.1	4.5	Wall-associated receptor kinase 5
TraesCS1B02G075700	8.4	14.8	40.1	Wall-associated receptor kinase 2

M30C24H vs MCK: DEGs in the infested Molly plants under heat stress.

N20C24H vs NCK: DEGs in the infested Newton under normal temperature.

N30C24H vs NCK: DEGs in the infested Newton under heat stress.

**Table 7:** Co-upregulated genes encoding defense proteins in susceptible newton and heat-induced susceptible molly plants at 24 h after initial HF attack.

Gene ID	Fold change			Gene description
	M30C24H vs MCK	N20C24H vs NCK	N30C24H vs NCK	
TraesCS2B02G369000	12.9	5.9	7.0	Chitinase 4/Chitin recognition protein
TraesCS2A02G350700	8.9	6.4	6.5	Chitinase 4/Chitin recognition protein
TraesCS2D02G348800	6.6	7.7	8.5	Chitinase 4/Chitin recognition protein
TraesCS7D02G454000	5.8	64.1	115.5	Chitin recognition protein
TraesCS7A02G466400	6.4	133.4	195.3	Chitin recognition protein
TraesCS5B02G261700	8.3	7.1	22.3	Thionin-like protein
TraesCS2B02G056700	2.0	6.2	9.7	Jacalin-like lectin domain
TraesCS1B02G314000	2.9	7.4	5.5	Protein LURP-one-related 8
TraesCS7A02G020900	4.3	3.0	3.9	Aspartic proteinase nepenthesin-1

M30C24H vs MCK: DEGs in the infested Molly plants under heat stress.

N20C24H vs NCK: DEGs in the infested Newton under normal temperature.

N30C24H vs NCK: DEGs in the infested Newton under heat stress.

**Table 8:** Co-upregulated genes encoding proteins with functions in secondary metabolism and structural defense in susceptible newton and heat-induced susceptible molly plants at 24 h after initial HF attack.

Gene ID	Fold change			Gene description
	M30C24H vs MCK	N20C24H vs NCK	N30C24H vs NCK	
TraesCS7B02G024400	5.2	22.5	34.8	Dirigent protein
TraesCS2D02G050200	19.7	4.5	11.3	Dirigent protein
TraesCS2D02G392800	20.8	77.9	256.6	Flavanone 3-dioxygenase 2
TraesCS2D02G317000	2.3	4.3	4.4	Glycosyltransferase
TraesCS1D02G350200	3.6	6.0	7.6	Glycosyltransferase

M30C24H vs MCK: DEGs in the infested Molly plants under heat stress.

N20C24H vs NCK: DEGs in the infested Newton under normal temperature.

N30C24H vs NCK: DEGs in the infested Newton under heat stress.

**Table 9:** Co-upregulated genes involved in oxidative stress in susceptible newton and heat-induced susceptible molly plants at 24 h after initial HF attack.

Gene ID	Fold change			Gene description
	M30C24H vs MCK	N20C24H vs NCK	N30C24H vs NCK	
TraesCS7A02G339600	5.3	26.2	11.9	Peroxidase, type III
TraesCS3D02G305300	4.5	28.5	14.3	Peroxidase, type III
TraesCS2A02G548200	2.2	2.1	3.9	Polyamine oxidase

M30C24H vs MCK: DEGs in the infested Molly plants under heat stress.

N20C24H vs NCK: DEGs in the infested Newton under normal temperature.

N30C24H vs NCK: DEGs in the infested Newton under heat stress.

**Table 10:** Co-upregulated genes encoding nutrient transporters in susceptible newton and heat-induced susceptible molly plants at 24 h after initial HF attack.

Gene ID	Fold change			Gene description
	M30C24H vs MCK	N20C24H vs NCK	N30C24H vs NCK	
TraesCS2A02G365000	5.9	23.7	11.1	Ammonium transporter
TraesCS5B02G470100	5.4	3.5	5.4	Probable inorganic phosphate/Sugar transporter
TraesCS7B02G213000	2.3	4.9	7.5	Amino acid transporter
TraesCS3A02G285100	4.1	3.7	10.4	ABC-2 type transporter

M30C24H vs MCK: DEGs in the infested Molly plants under heat stress.

N20C24H vs NCK: DEGs in the infested Newton under normal temperature.

N30C24H vs NCK: DEGs in the infested Newton under heat stress.

**Table 11:** Other co-upregulated genes involved in stress response and developmental regulation in susceptible newton and heat-induced susceptible molly plants at 24 h after initial HF attack.

Gene ID	Fold change			Gene description
	M30C24H vs MCK	N20C24H vs NCK	N30C24H vs NCK	
TraesCS3A02G333500	4.4	2.3	3.8	F-box protein
TraesCS3B02G364700	3.5	2.6	3.0	F-box protein
TraesCS3D02G327100	3.8	2.5	2.8	F-box protein
TraesCS5D02G371500	2.9	55.9	103.6	Alpha/beta hydrolase family
TraesCS4A02G247500	4.0	3.1	2.4	GEM-like protein 1/GRAM domain
TraesCS1A02G035600	8.3	9.9	14.3	Cysteine-rich repeat secretory protein 38
TraesCS3B02G306600	2.3	2.5	3.0	GDL82_ GDSL esterase/lipase

M30C24H vs MCK: DEGs in the infested Molly plants under heat stress.

N20C24H vs NCK: DEGs in the infested Newton under normal temperature.

N30C24H vs NCK: DEGs in the infested Newton under heat stress.

**Table 12:** Commonly downregulated DEGs in susceptible newton and heat-induced susceptible molly plants at 24 h after initial HF attack.

Gene ID	Fold change			Gene description
	M30C24H vs MCK	N20C24H vs NCK	N30C24H vs NCK	
Defense-related secondary metabolism				
TraesCS2B02G031600	0.088	0.429	0.046	α-terpineol synthase, chloroplastic
TraesCS2D02G023600	0.164	0.079	0.058	α-terpineol synthase, chloroplastic
TraesCS2D02G023700	0.225	0.111	0.021	Indole-2-monooxygenase
TraesCS5A02G553100	0.048	0.143	0.011	Deoxyloganetin glucosyltransferase
Lipid metabolism and defense signalling				
TraesCS1B02G367500	0.230	0.429	0.240	Phospholipase A1
TraesCS1B02G367700	0.316	0.480	0.245	Phospholipase A1
Water transport and cell wall metabolism				
TraesCS6D02G076400	0.497	0.227	0.137	Aquaporin PIP2-4
TraesCS7D02G360100	0.394	0.461	0.259	Xyloglucan endotransglucosylase/hydrolase
Programmed cell death and hypersensitive responses				
TraesCS1A02G404600	0.323	0.189	0.015	Subtilisin-like protease
TraesCS3D02G534200	0.363	0.348	0.166	Vacuolar-processing enzyme gamma-isozyme
TraesCS5B02G059300	0.424	0.369	0.225	Non-specific serine/threonine protein kinase

M30C24H vs MCK: DEGs in the infested Molly plants under heat stress.

N20C24H vs NCK: DEGs in the infested Newton under normal temperature.

N30C24H vs NCK: DEGs in the infested Newton under heat stress.

#### Co-upregulated phytohormone-related genes

Twenty-two of 63 (35%) co-upregulated genes were directly linked to phytohormone signaling, metabolism, or regulation (**Table 5**). Eighteen of these genes are involved in auxin perception, signaling, and homeostasis, one gene (ethylene-responsive transcription factor ABR1/AP2 domain) is involved ethylene signaling, one gene (lipoxygenase) is involved in the 12-oxo-phytodienoic acid (OPDA)/jasmonic acid (JA) pathway, and two genes (DMR6-like oxygenase 1) are involved in salicylic acid (SA) metabolism. Auxin related genes include auxin-repressed protein, auxin responsive factor/protein, indole-3-acetic acid-amido synthetase functioning in converting active IAA into inactive conjugates (Bao et al., 2024; Casanova‐Sáez et al., 2022), VAN3-binding protein functioning in auxin canalization (Sawa et al., 2005), and flavin-containing monooxygenase, a key enzyme in auxin biosynthesis (Luo and Di, 2023). Notably, all three-auxin repressed protein exhibited extremely high induction with over 200-fold change in the heat-stressed Molly and Newton plants, which is much higher than the fold change observed in the Newton plants under normal temperature (**Table 5**). The flavin-containing monooxygenase, ethylene-responsive transcription factor ABR1/AP2 domain, and one of the two DMR6-like oxygenase (TraesCS2D02G392900), the negative regulators of SA accumulation (Pirrello et al., 2022), exhibited much higher induction in the susceptible Newton plants under both normal and heat-stressed conditions compared to that in the partially susceptible Molly plants under heat conditions.

#### Co-upregulated genes encoding transcription factors and cell regulators

Thirteen genes consistently upregulated in all susceptible conditions belonged to the category of transcription factors and signaling regulators (**Table 6**). Four of the genes encode for WRKY transcription factors (WRKY70, WRKY1, WRKY45-like), which regulate a wide range of physiological processes including phytohormone signaling and cross-talking. For example, WRKY70 is induced by SA and repressed by JA. Overexpression of WRKY70 boosts SA-dependent defenses and suppresses JA-responsive genes (Li et al., 2004). WRKY45 mediates SA-mediated resistance in cereals (Shimono et al., 2007). Three genes including WUSCHEL-related homeobox 7, a B-box zinc finger protein, and a cell number regulator 2, encode developmental regulators (**Table 6**). These genes coordinate developmental processes like growth, stress responses, and meristem activity by regulating cell division and communication (Kong et al., 2016; Talar and Kiełbowicz-Matuk, 2021; Beauchet et al., 2024). Six genes encode receptor-like kinases, including two LRR receptor-like serine/threonine-protein kinase, one G-type lectin S-receptor-like serine/threonine-protein kinase, and three wall-associated kinases. Receptor-like kinases function as plasma-membrane receptors that perceive extracellular or cell wall-derived signals and activate downstream signaling cascades (Ma et al., 2024). Overall, the increased transcript abundance of these genes underscores the activation of high-level regulatory and signaling systems.

#### Co-upregulated genes encoding defense proteins

This category includes genes encoding well-established defense-related proteins that are widely associated with antimicrobial activity, cell wall fortification and immune signaling (**Table 7**). This group includes three chitinase/chitin-recognition proteins, two chitin-recognition proteins, a thionin-like protein, a jacalin-like lectin, a LURP-one-related 8, and an aspartic proteinase nepenthesin-1. The fold changes of the chitin recognition proteins in Newton plants with or without heat stress were much higher than that in the heat-stressed Molly plants.

#### Co-upregulated genes encoding proteins with functions in secondary metabolism and structural defense

This group includes genes encoding dirigent proteins, flavanone 3-dioxygenase, and glycosyltransferases (**Table 8**), which are involved in lignin biosynthesis (Paniagua et al., 2017), flavonoid biosynthesis (Hammerbacher et al., 2019), and cell wall remodeling (Barnes and Anderson, 2018). Those are all key processes in reinforcing physical barriers against insect invasion. The upregulation of a dirigent gene (TraesCS7B02G024400), and the flavanone 3-dioxygenase 2 gene were much higher in the Newton plants with or without heat stress than in the heat-stressed Molly plants (**Table 8**).

#### Co-upregulated genes involved in oxidative stress

This category includes two type III peroxidases and a polyamine oxidase (**Table 9**), which play key roles in scavenging hydrogen peroxide and maintaining ROS (reactive oxygen species) balance during stress responses, contributing to cell wall strengthening and signaling under pathogen/insect attack (Angelini et al., 2008; Almagro et al., 2009; Minibayeva et al., 2015), The upregulation of these two peroxidases was apparently higher in Newton plants than that in the Molly with the highest upregulation in the susceptible Newton plants under normal temperature.

#### Co-upregulated genes encoding nutrient transporters

This category includes genes encoding transporters involved in the uptake and redistribution of key nutrients, which include an ammonium transporter, a phosphate/sugar transporter, an amino acid transporter, and an ABC-2 type transporter (**Table 10**). These transporters mediate nitrogen, phosphorus, and amino acid fluxes, as well as the translocation of various metabolites and signaling molecules (Liu et al., 1998; Do et al., 2018; Hao et al., 2020; Yang et al., 2020).

#### Other co-upregulated genes involved in stress responses and development

Additionally, seven co-upregulated genes in susceptible plants fell outside the major functional categories but are associated with development, hormone signaling, and stress adaptation (**Table 11**). These included three F-box proteins involved in ubiquitin-mediated regulation of hormonal and defense pathways (Dharmasiri et al., 2005), an alpha/beta hydrolase strongly induced in Newton, a GEM-like/GRAM domain protein that are ABC-responsive regulators (Mauri et al., 2016), a cysteine-rich secretory protein functions in development and stress adaptation (Matsuzawa et al., 2024), and a GDSL esterase/lipase with diverse function including lipid metabolism, growth and development, and stress responses (Duan et al., 2023).

#### Genes downregulated in susceptible Newton and heat-induced susceptible Molly plants

A set of 11 genes was consistently downregulated in all HF-infested susceptible Newton plants and heat-induced susceptible Molly plants as well as Newton plants under heat stress (**Table 12**). The co-downregulated genes span four major functional categories. The first category comprises four genes involved in defense-related secondary metabolism, including two α-terpineol synthases that contribute to terpenoid-based defenses, an indole-2-monooxygenase, a key enzyme in the biosynthetic pathway of benzoxazinoids such as DIMBOA-glucoside that deter insect herbivores (Frey et al., 2009), and a deoxyloganetin glucosyltransferase, an enzyme associated with biosynthesis of iridoids, which are compounds with well-established insecticidal functions in plants (Asada et al., 2013). The second category comprises two phospholipase A1 (PLA1) genes involved in lipid metabolism and defense signaling. PLA1 enzymes are known to participate in OPDA/JA biosynthesis and membrane remodeling that is critical for defense against insect herbivores, including HF (Yang et al., 2012). The third category comprises aquaporin PIP2–4 and a xyloglucan endotransglucosylase that function in water transport and cell wall metabolism. Aquaporin PIP2–4 regulates water transport and maintains cell turgor (Maurel et al., 2015), and xyloglucan endotransglucosylase modifies cell wall components and strengthens cell wall defense (Rose et al., 2002). The fourth category comprises three genes involved in programmed cell death and hypersensitive responses, which include a subtilisin-like protease, a vacuolar-processing enzyme gamma-isozyme and a non-specific serine/threonine protein kinase. Hypersensitive responses is a critical defense mechanism limiting insect feeding sites through programmed cell death (Balint-Kurti, 2019). Collectively, these co-downregulated genes highlight a core set of defense-related pathways suppressed in the susceptible Newton under normal and heat-stressed conditions and in the heat-stressed Molly whose resistance was compromised.

## Discussion

Our study demonstrates that a single day of heat stress at 30 °C is sufficient to convert the HF-resistant wheat cultivar Molly into a susceptible phenotype, with no further increase in susceptibility observed for heat stresses of 48 or 96 hours (**Figure 1**). This implies that a critical physiological threshold is reached within the first 24 h of heat stress. Consistent with these phenotyping results, transcriptome profiling revealed that combined heat stress and HF infestation triggered extensive transcriptional reprogramming in Molly plants with the number of DEGs nearly three times higher than that in the naturally susceptible Newton line and 13 times higher than in resistant Molly plants under normal temperature (**Figures 4**, **5**). This large-scale reprogramming reflects the extent to which heat stress destabilizes normally effective resistance mechanisms and rewrites plant immunity, as has been observed in wheat-HF interaction and plant interaction with pathogens (Cohen et al., 2017; Zhu et al., 2020; Yuan et al., 2022; Shelake et al., 2024).

### Hormonal crosstalk and auxin-associated susceptibility

Among the most prominent susceptibility-associated genes were those involved in auxin perception, transport, and homeostasis. Three genes encoding auxin-repressed proteins showed fold-changes exceeding 200–400 in heat-stressed Molly and Newton, suggesting substantial changes in auxin-related transcriptional responses under susceptible conditions. Additional induction of auxin-responsive factors, GH3 family indole-3-acetic acid (IAA)-amido synthetases, and VAN3-binding proteins suggests coordinated modulation of auxin canalization and conjugation (Staswick et al., 2005; Teale et al., 2006; Xu et al., 2019). At the same time, a flavin-containing monooxygenase, an enzyme in IAA biosynthesis (Cheng et al., 2006) was strongly upregulated (**Table 5**). The upregulation of these auxin associated genes is consistent with our previous finding indicating the significantly increased auxin accumulation in the compatible wheat-HF interaction (Zhu et al., 2010). Auxin accumulation is known to antagonize SA-mediated plant immunity, often promoting pathogen and insect virulence (Kazan and Manners, 2009). Consistent with this, we observed co-upregulation of two DMR6-like oxygenase genes (**Table 5**), which are negative regulators of SA accumulation (Thomazella et al., 2021). SA mediated defense responses are essential for wheat resistance to HF, as evidenced by the significantly increased accumulation of SA observed during the incompatible wheat-HF interaction (Zhu et al., 2010, 2011). Additionally, our previous studies have shown that exogenous application of SA solution reduces HF larval survival at feeding sites under normal temperature and enhanced wheat resistance to HF under heat stress (Zhu et al., 2012; Underwood et al., 2014). Further, we observed downregulation of two phospholipase A1 (PLA1) genes, which are involved in lipid signaling and initiation of OPDA biosynthesis in the susceptible plants. This observation is consistent with our earlier reports showing that high OPDA accumulation may contribute to wheat resistance to HF, and that heat stress decreases OPDA accumulation at the HF feeding sites (Zhu et al., 2010, 2011; Currie et al., 2014b). Taken together, these findings suggest that auxin-associated transcriptional responses, along with reduced SA- and OPDA-related signaling, are associated with susceptibility in both heat-stressed Molly and Newton plants.

### Suppression of core defense pathways

In parallel with the hormonal patterns described above, 11 genes were consistently downregulated in both susceptible Newton and heat-induced susceptible Molly plants, reflecting reduced defense-responses(**Table 12**). Several of these genes are involved in secondary metabolism, including two α-terpineol synthases, an indole-2-monooxygenase required for benzoxazinoid biosynthesis, and a deoxyloganetin glucosyltransferase linked to iridoid production. These metabolic pathways are known to contribute to chemical defenses that deter herbivory (Frey et al., 2009). Additional downregulation was observed in aquaporin PIP2–4 and a xyloglucan endotransglucosylase, suggesting potential disruption of water balance and impaired cell wall reinforcement (Huang et al., 2012). Likewise, the downregulation of a subtilisin-like protease, a vacuolar-processing enzyme, and a non-specific serine/threonine kinase points to weakened hypersensitive response and programmed cell death, two processes that normally function to restrict larval feeding sites (Hatsugai et al., 2015; Figueiredo et al., 2017). The coordinated downregulation of these genes across multiple defense pathways, including secondary metabolism, water transport, cell wall remodeling, and localized cell death suggests that susceptibility is likely associated with reduced activation or coordination of defense-related processes in both naturally susceptible and heat stressed wheat plants.

### Defense-related transcriptional reprogramming and host manipulation in susceptible plants

One of the most notable outcomes of this study is that many genes with annotated defense-related functions were strongly induced in all susceptible states including Newton under both normal temperature and heat stress and Molly under heat stress,. however, their expression remained unchanged in resistant Molly under normal conditions (**Table 5**-11). These induced genes included classical defense regulators such as WRKY70 and WRKY45-like transcription factors, receptor-like kinases such as wall-associated kinases, developmental regulators such as cell number regulator 2 and WUSCHEL-related homeobox 7 (**Table 6**), and enzymes involved in secondary metabolism including dirigent proteins and flavanone 3-dioxygenase 2 (**Table 8**). Antimicrobial proteins such as chitinase 4, thionin-like proteins, and jacalin-like lectins (**Table 7**), as well as oxidative stress related enzymes such as peroxidases and polyamine oxidase (**Table 9**) were also consistently upregulated. Despite their classical association with plant resistance responses, none of these genes were induced in the resistant Molly, which kills HF larvae before they enter second instar (**Figure 1**). In susceptible plants, virulent HF larvae induce the formation of nutritive tissue at the feeding site, altering the attacked wheat tissue into a localized metabolic sink that supports larval growth and development (Harris et al., 2006). Previous studies have shown that HF attack causes major shifts in carbon and nitrogen metabolism in wheat and that virulent larvae alter the free amino acid and polyamine content of host wheat plants at feeding sites, further supporting the hypothesis that susceptible interactions involve the generation of a nutritionally favorable environment for larval development (Saltzmann et al., 2008; Zhu et al., 2008; Subramanyam et al., 2015). From this perspective, the defense-related genes induced here in susceptible Newton and heat-induced susceptible Molly may not simply represent unsuccessful or insufficient resistance responses but may also reflect physiological changes in the host that are associated with susceptibility and feeding-site establishment. This interpretation is further supported by the co-upregulation of nutrient transporter genes in all susceptible plants in this study, including genes encoding ammonium, phosphate/sugar, amino acid, and ABC-type transporters (**Table 10**). The induction of these transporters is consistent with increased nutrient mobilization and redirection toward the feeding site. Increased expression of ammonium and amino acid transporters may facilitate nitrogen remobilization, whereas the phosphate/sugar transporter may contribute to carbohydrate and phosphorus delivery to developing nutritive tissues (Liu et al., 1998; Hao et al., 2020; Yang et al., 2020). Meanwhile, increased expression of ABC-type transporters may further support the movement of metabolites or signaling molecules required to maintain the altered physiological state of susceptible tissues (Do et al., 2018).

To summarize, this study demonstrates that heat stress rapidly compromises *H13*-mediated resistance in wheat and induces transcriptional reprogramming associated with susceptibility to HF. Comparative transcriptomic analysis identified a set of genes consistently regulated across susceptible states. Susceptibility in naturally susceptible Newton wheat and heat-stressed Molly wheat is associated with increased auxin-related activity, reduced SA- and OPDA-mediated defense signaling, suppressed core defense-related pathways, which may favor HF feeding. The induction of many defense related genes in all susceptible plants, but not in resistant plants suggests that despite the induction of defense-related genes, they are not sufficient for triggering an effective HF resistance. The identification of susceptibility-associated candidate genes provides molecular targets for breeding wheat cultivars with more durable resistance under rising temperatures.

## Data Availability

Data have been deposited in the NCBI BioProject repository (https://www.ncbi.nlm.nih.gov/), accession number: PRJNA1406795.
