# Supplementary material for: Transcriptomic changes associated with heat-induced susceptibility in wheat plants to Hessian fly (Diptera: Cecidomyiidae)
Source: Front Plant Sci. 2026 May 5;17:1794823. doi: 10.3389/fpls.2026.1794823 (PMC13184837; doi:10.3389/fpls.2026.1794823)
Supplement: Supplementary file 1 [file Table1.docx]

Supplementary Table 1**.**  Accession number, annotated gene name abbreviation, and primers used for qRT-PCR validation.

| **Accession** | **Gene abbreviation** | **Forward primer**  **5’-3’** | **Reverse primer**  **5’-3’** |
| --- | --- | --- | --- |
| TraesCS2A02G548200 | PAO | CACGGCGCCTACTCATCTG | AACGCCCTTACTCATCAGCAA |
| TraesCS2D02G065200 | RBC | ACAAACACGCGGACCATTG | GACATATTCATTGCAGTGCCAAA |
| TraesCS3B02G390700 | HSP | TGGCCGTGGTGATTTGG | TCACAAACTTAACACTCCAGAGTTCA |
| TraesCS2D02G033200 | HSP | TGGTCGCTGCGTGTCAAG | CCCGAGAGTCAAACAAGAAGGA |
| TraesCS2A02G033700 | HSP | GTGAGTGAGTTCTAGTGGTTGTTGGT | TTGACCCGAGAATGAAACAAGA |
| TraesCS4D02G069100 | ARP | GAAGACAGGGTGCTCAACAACTT | CGCTATGCAGGATTAGATAACCAA |
| TraesCS7D02G417400 | POX | GCAAATGTGGCATGGTCATG | GCAAATGTGGCATGGTCATG |
| TraesCS1A02G266100 | PLA1 | GCAGAGTGGTGGGTCCCTAA | TGCCCATCGTCCATCCTT |
| TraesCS4B02G212300 | HSP | GAGCAGAGGTGTGGAGTTTCG | CGGCCGGACTCTGGTTT |
| TraesCS4B02G070300 | ARP | TTTGTCTTGAGCTTCGTACTACTGTCTA | TGAGCAGACCGGGATAAGAAA |
| TraesCS2B02G574800 | FMOs | TGATGCATTGGAGGGACAACT | CAAAGCCCAGGGCCAAA |
| TraesCS7D02G199400 | CAB | GCATTTGCCACCAACTTCGT | CCCGCCAGCTCTCAACTC |
| TraesCS1A02G013600 | Defensin | CCACTGACAATTTGGCTTCTATGT | ACAAGCCAACAAACCTGCTGAT |
| TraesCS4A02G149800 | GTPase | CGCCTACTGTGGCGTAGCA | GCTTCCGTATTTGATCTCCTCTGT |
| TraesCS2A02G544500 | FMO | ATGCCTCGCGGAAGAACAC | CATAGTGGCGAGAGATGTTTCG |
| TraesCS4B02G021300 | PK | CAGCATGGACGACAACTTCAA | CACCGAGCCCTTGGACTTC |
| TraesCS2A02G313500 | Oxoacyl-syn | GGTAGAGTGGTGCTGCCTTGA | GGAAGCACCCGAAAGAATTACTG |
| TraesCS5B02G462800 | CAB | GGCAACCCAAGCCTTGTG | ACGACCTGACAAGCCCAGAT |
| TraesCS1A02G427200 | PTAC14 | TCCGACGAGCCTATGAGGAA | GGTGCAGCCGGTACTTGATC |
| TraesCS6D02G145700 | RNA-MT | GCTACAAAGTGCATCCGGTATG | CCCTGGCTTTACCACTTGCAT |

PAO: Polyamine oxidase

RBC: Ribulose bisphosphate carboxylase

HSP: Heat shock protein

ARP: Auxin-repressed protein

POX: Peroxidase

PLA: Phospholipase A1

ARP: Auxin-repressed protein

FMOs: Flavin-containing monooxygenases

CAB: Chlorophyll a-b binding protein

PK: Pyruvate kinase

Oxoacyl-syn: 3-Oxoacyl synthase

PTAC14: Plastid transcriptionally active 14

RNA-MT: Ribosomal RNA methyltransferase
